# Qualitative and quantitative differences between taste buds of the rat and mouse

**DOI:** 10.1186/1471-2202-8-5

**Published:** 2007-01-05

**Authors:** Huazhi Ma, Ruibiao Yang, Stacey M Thomas, John C Kinnamon

**Affiliations:** 1 Department of Biological Sciences, University of Denver, Denver, Colorado 80210, USA; 2 Rocky Mountain Taste and Smell Center, Aurora, Colorado 80045, USA; 3 Beijing Institute of Pharmacology and Toxicology, Beijing 100850, China

## Abstract

**Background:**

Numerous electrophysiological, ultrastructural, and immunocytochemical studies on rodent taste buds have been carried out on rat taste buds. In recent years, however, the mouse has become the species of choice for molecular and other studies on sensory transduction in taste buds. Do rat and mouse taste buds have the same cell types, sensory transduction markers and synaptic proteins? In the present study we have used antisera directed against PLCβ2, α-gustducin, serotonin (5-HT), PGP 9.5 and synaptobrevin-2 to determine the percentages of taste cells expressing these markers in taste buds in both rodent species. We also determined the numbers of taste cells in the taste buds as well as taste bud volume.

**Results:**

There are significant differences (p < 0.05) between mouse and rat taste buds in the percentages of taste cells displaying immunoreactivity for all five markers. Rat taste buds display significantly more immunoreactivity than mice for PLCβ2 (31.8% vs 19.6%), α-gustducin (18% vs 14.6%), and synaptobrevin-2 (31.2% vs 26.3%). Mice, however, have more cells that display immunoreactivity to 5-HT (15.9% vs 13.7%) and PGP 9.5 (14.3% vs 9.4%). Mouse taste buds contain an average of 85.8 taste cells vs 68.4 taste cells in rat taste buds. The average volume of a mouse taste bud (42,000 μm^3^) is smaller than a rat taste bud (64,200 μm^3^). The numerical density of taste cells in mouse circumvallate taste buds (2.1 cells/1000 μm^3^) is significantly higher than that in the rat (1.2 cells/1000 μm^3^).

**Conclusion:**

These results suggest that rats and mice differ significantly in the percentages of taste cells expressing signaling molecules. We speculate that these observed dissimilarities may reflect differences in their gustatory processing.

## Background

Mammalian taste buds are onion-shaped structures specialized for the detection of aqueous stimuli. Based on morphological criteria, rodent taste cells have been classified into types I, II, III, peripheral and basal cells [1-12]. Type I cells in rodents are slender and possess an electron-dense cytoplasm and several long, apical microvilli extending into the oral cavity. A distinguishing feature of a type I cell is the presence of many 100–400 nm dense granules in the apical cytoplasm. Type II cells are characterized by the presence of an electron-lucent cytoplasm and large circular or ovoid nuclei. Type II cells possess several short microvilli of uniform length extending into the taste pore. Type III cells are slender and exhibit morphology and cytoplasmic electron density intermediate between type I and type II cells. The nuclei of type III cells are slender and possess prominent invaginations. Two distinguishing features of type III cells are the single blunt microvillus that extends into the taste pore and the presence of synapses onto nerve processes [11,13,14].

Only recently are the functional differences of the cell types becoming understood. Still, it is not clear which taste cell types are the receptors. Based on the presence of synaptic foci, it was believed that type III cells were the only taste bud receptor cells [15-18]. Evidence that type II cells are associated with transduction molecules, however, suggested a sensory for this cell type. For example, some type II taste cells express the taste signaling molecules α-gustducin, PLCβ2, and the type III IP_3 _receptor (IP_3_R3) in rat circumvallate taste buds [19-22]. It is significant, however, that type II taste cells apparently lack classical synapses. Likewise, some type III taste cells display immunoreactivity to serotonin (5-HT) in rat and mouse circumvallate taste buds [23], neural cell adhesion molecule (NCAM) [24], and synaptosome-associated protein of 25 kDa (SNAP-25) in rat circumvallate taste buds [13]. Immunoreactivity to ubiquitin carboxyl terminase (protein gene product 9.5, [PGP 9.5]) [11] and the synaptobrevin-2 (vesicle associated membrane protein-2, VAMP-2) [14] are both found in type II and III taste cells in rat circumvallate taste buds. A small percentage (3.5%) of PLCβ2 or IP_3_R3 immunoreactive cells also display 5-HT-LIR. It is believed that PLCβ2 or IP_3_R3 is also present in a small subset of type III cells in rat circumvallate taste buds [21]. Quantitation studies have demonstrated that approximately 24% of the taste cells in rat circumvallate papillae display α-gustducin-LIR [25], whereas another study showed that α-gustducin is present in 33% of taste cells in mouse circumvallate papillae [26]. PGP 9.5 is present approximately in 14.6% of the taste cells in rat circumvallate taste buds [25] and 23% of taste cells in mouse circumvallate taste buds [26]. Based on these preliminary data, it is likely that there are differences in cell type labeling between rats and mice.

Many of the electrophysiological, ultrastructural, and immunocytochemical studies on rodent taste buds have been carried out on rat taste buds. In recent years, however, the mouse has become the species of choice for molecular and other studies on sensory transduction in taste buds. Do rat and mouse taste buds have the same cell types, sensory transduction markers and synaptic proteins? Recent research indicates that there are differences in electrophysiological properties, expression of markers and innervation between rat and mouse taste buds [27-30]. The acid-sensing ion channel-2 (ASIC-2) is widely believed to be a receptor for acid taste in rat taste cells, however, ASIC-2 is not expressed in mouse taste cells and ASIC-2 knock-out mice exhibited normal physiological responses to acid taste stimuli [28]. ASIC-2 is an acid taste receptor in rat taste cells, but not in mouse taste cells. Rat and mouse taste buds are innervated differently by peripheral taste neurons [29,30]. Three to five ganglion cells innervate a single bud in mice while there is a more divergent innervation of buds in the rat [29,30]

In the present study we have used antisera directed against PLCβ2, α-gustducin, 5-HT, PGP 9.5 and synaptobrevin-2 to determine the percentages of taste cells expressing these markers in circumvallate taste buds of both rodent species. In addition we have determined the numerical density of taste cells and taste bud volume between rat and mouse circumvallate taste buds using serial transverse sections.

## Results

### Serotonin (5-HT)

Serotonin-LIR is present in a small subset of taste cells in rodent taste buds. The animal is injected with the immediate precursor, 5-HTP, according to the method of Kim and Roper [23]. Previous studies have demonstrated that serotonin is present in a subset of type III taste cells in rat and mouse circumvallate taste buds [11,23]. Our results show that a small subset of slender taste cells display serotonin-like immunoreactivity (LIR) in both rat and mouse circumvallate taste buds.

**Figure 1:**
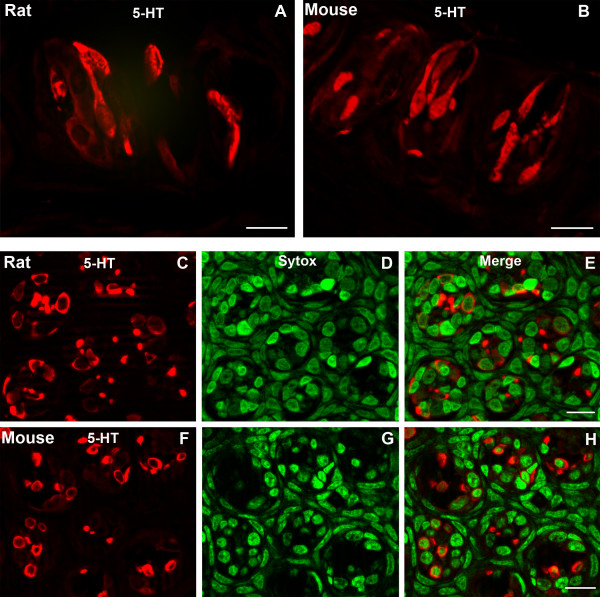
Confocal laser scanning microscopy (CLSM) images of 5-HT-LIR (5-HT) in taste buds of rat and mouse circumvallate papillae. Longitudinal sections show a small subset of taste cells displaying 5-HT-LIR in rat (A) and mouse taste buds (B). Both cytoplasm and nuclei of taste cells display 5-HT-LIR. Transverse sections show 5-HT-LIR in rat (C-E) and mouse taste buds (F-H). The red cells are immunoreactive taste cells (C and F). Sytox-stained nuclei are shown in green (D and G), which stain all cells. Merges of red and green images are shown in E and H. Scale bars = 20 μm.

**Figure 2:**
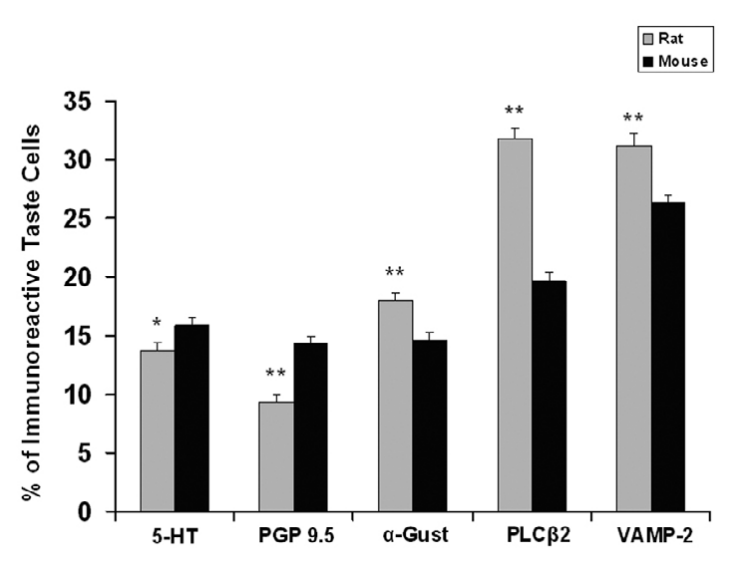
Quantitation of taste cells displaying different immunoreactivity to markers in rat and mouse circumvallate taste buds. Values represent the mean percentages of immunoreactive taste cells ± SEM. *, p < 0.05; **, p < 0.01. α-Gust, α-gustducin; VAMP-2, synaptobrevin-2.

Immunoreactivity is present in both the cytoplasm and nuclei (Fig. 1). A single taste bud profile contains approximately 2.5 taste cells in rat and 2.8 taste cells in mouse displaying serotonin immunoreactivity (Table 1). We examined 141 taste buds from 5 rats and 221 taste buds from 10 mice. A total of 353 immunoreactive cells were found in the rat taste buds and 621 immunoreactive cells in the mouse taste buds were counted. There is a significant difference between rat (13.7%) and mouse circumvallate taste buds (15.9%) in the percentage of taste cells displaying serotonin-LIR (p < 0.05) (Fig. 2).

**Table 1:** Quantitation of immunoreactive cells in rat and mouse circumvallate taste buds

**Antibodies**	**Animals**	**No. of TBP**	**No. of TCs**	**TCs/TBP (Mean ± SD)**	**No. of LIR-TCs**	**LIR-TCs/TBP (Mean ± SD)**
5-HT	5 rats	141	2681	19.0 ± 6.5	353	2.5 ± 1.5
	10 mice	221	4019	18.2 ± 6.1	621	2.8 ± 1.6
PGP 9.5	5 rats	144	2617	18.2 ± 5.9	240	1.7 ± 1.2
	10 mice	146	3449	23.6 ± 6.8	437	3.0 ± 1.5
α-gustducin	5 rats	197	3757	19.1 ± 6.9	635	3.6 ± 1.5
	10 mice	181	3619	20.0 ± 7.0	482	2.7 ± 1.5
PLCβ2	5 rats	152	2935	19.3 ± 7.3	935	6.2 ± 3.1
	10 mice	163	3549	21.8 ± 8.2	666	4.1 ± 2.1
VAMP-2	5 rats	151	2910	19.3 ± 6.6	870	5.8 ± 2.2
	10 mice	241	5063	21.0 ± 7.0	1290	5.4 ± 2.4

TCs = Taste cell, LIR-TCs = Immunoreactive taste cells, TBP = Taste bud profile.

### PGP 9.5

**Figure 3:**
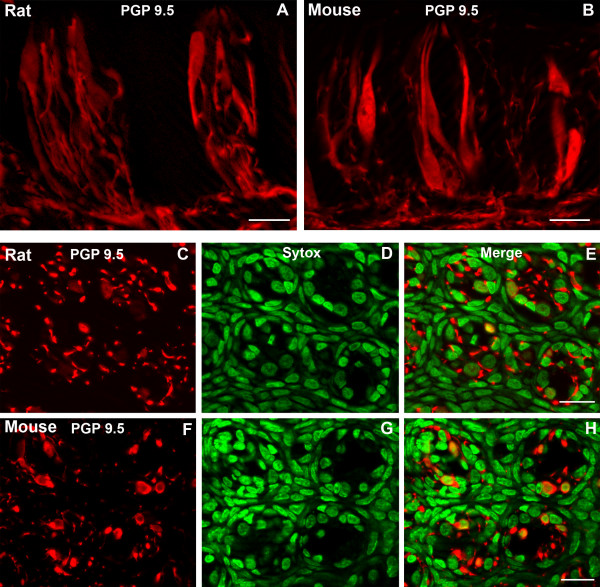
Confocal laser scanning microscopy (CLSM) images of PGP 9.5-LIR (PGP 9.5) in taste buds of rat and mouse circumvallate papillae. Longitudinal sections show a small subset of taste cells and nerve processes expressing PGP 9.5 in rat (A) and mouse taste buds (B). Both cytoplasm and nuclei of taste cells display PGP 9.5-LIR. Transverse sections show PGP 9.5-LIR in rat (C-E) and mouse taste buds (F-H). Immunoreactive taste cells and nerve processes are shown in C and F. Sytox-stained nuclei are shown in D and G and merges are shown in E and H. Scale bars = 20 μm.

Subsets of taste cells and nerve processes in both rat and mouse circumvallate taste buds display PGP 9.5-LIR (Fig. 3). Three subsets of PGP 9.5-LIR nerve processes are present: intragemmal, perigemmal and extragemmal. Intense immunoreactivity is associated with the nerve plexus located at the base of the taste bud. Some PGP 9.5-LIR taste cells are slender, spindle-shaped cells with irregular nuclei, while others have large ovoid to round nuclei. Whereas each taste bud profile in the rat contains approximately 1.7 PGP 9.5-LIR taste cells, approximately 3 taste cells per taste bud profile are immunoreactive for PGP 9.5 in the mouse (Table 1). There is a significant difference (p < 0.001) in the percentages of PGP 9.5 immunoreactive taste cells between rat and mouse circumvallate taste buds. Approximately 14.3% of the taste cells in the mouse display PGP 9.5-LIR, while only 9.4% taste cells in the rat exhibit PGP 9.5-LIR (Fig. 2).

### α-gustducin

α-gustducin is a G protein believed to be involved in the transduction pathways for bitter and sweet taste [31-34]. α-gustducin may also play a role in umami taste [35,36]. α-gustducin is present in a subset of type II cells [19]. Our results show that a subset of taste cells express α-gustducin-LIR in both mouse and rat circumvallate taste buds. The α-gustducin-LIR taste cells are spindle-shaped with large, round nuclei. Immunoreactivity is cytoplasmic; no immunoreactivity is associated with the nuclei.

**Figure 4:**
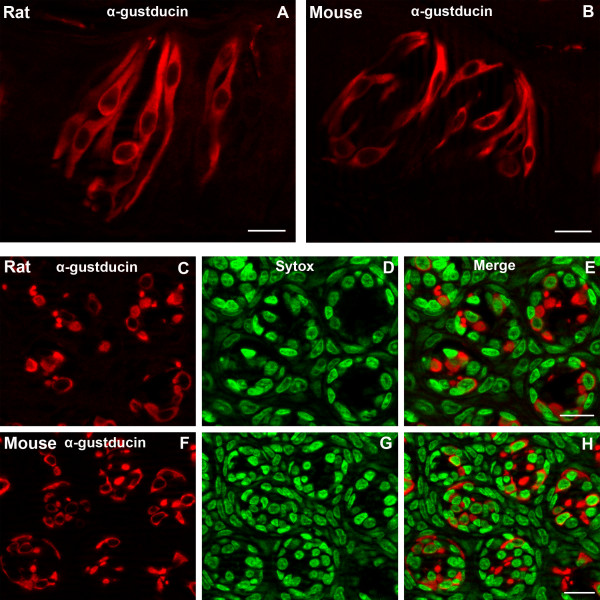
Confocal laser scanning microscopy (CLSM) images of α-gustducin-LIR (α-gustducin) in taste buds of rat and mouse circumvallate papillae. Longitudinal sections show a subset of taste cells displaying α-gustducin-LIR in rat (A) and mouse taste buds (B). The α-gustducin-LIR taste cells are spindle-shaped with large, round nuclei. Transverse sections show α-gustducin-LIR in rat (C-E) and mouse taste buds (F-H). The immunoreactivity is only cytoplasmic in both transverse and longitudinal sections. Immunoreactive taste cells (red) are shown in C and F. Sytox-stained nuclei (green) are shown in D and G and merges are shown in E and H. Scale bars = 20 μm.

α-gustducin immunoreactive cells extend from the basal lamina to the taste pore (Fig. 4). We analyzed 197 taste buds from five rats and 181 taste buds from ten mice. Cells were scored as immunoreactive only if the cellular profile contained a nuclear profile. We observed 635 immunoreactive taste cells in the rat and 482 immunoreactive taste cells in mouse taste buds (Table 1). Approximately 18% of the taste cells in rat taste buds and 14.6% of taste cells in mouse taste buds displayed α-gustducin-LIR. The numbers of α-gustducin-LIR immunoreactive taste cells in the rat were significantly different from those in the mouse (p < 0.01) (Fig. 2).

### PLCβ2

**Figure 5:**
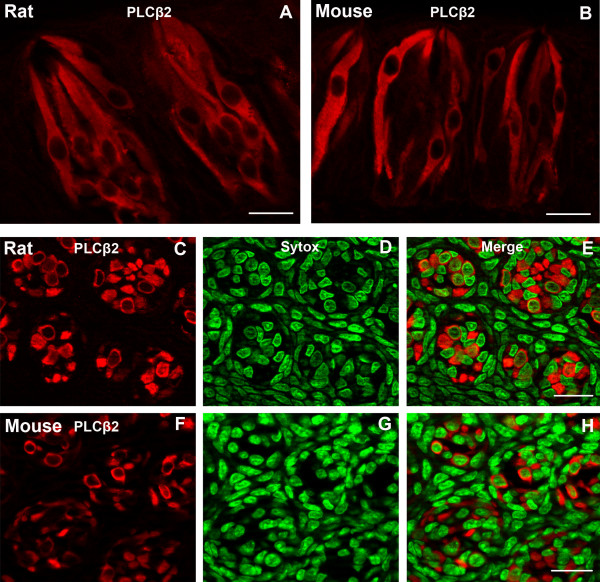
Confocal laser scanning microscopy (CLSM) images of PLCβ2-LIR (PLCβ2) in taste buds of rat or mouse circumvallate papillae. Longitudinal sections show a large subset of taste cells expressing PLCβ2 in rat (A) and mouse taste buds (B). The PLCβ2-LIR taste cells are spindle-shaped with large, round nuclei. Transverse sections show PLCβ2-LIR in rat (C-E) and mouse taste buds (F-H). The immunoreactivity is restricted to the cytoplasm in both transverse and longitudinal sections. Immunoreactive taste cells (red) are shown in C and F. Sytox-stained nuclei (green) are shown in D and G and merges are shown in E and H. Scale bars = 20 μm.

Phospholipase Cβ2 (PLCβ2) is thought to be essential for the transduction of bitter, sweet, and umami stimuli [37]. A large subset of taste cells in both rat and mouse circumvallate taste buds display PLCβ2-LIR. The immunoreactive cells are spindle-shaped with round nuclei resembling type II taste cells (Fig. 5). We counted 935 PLCβ2-LIR cells from 152 rat taste buds and 666 PLCβ2-LIR cells from 163 mouse taste buds. Whereas 31.8% of rat circumvallate taste cells display PLCβ2-LIR, only 19.6% of the mouse circumvallate taste cells display PLCβ2-LIR. Thus, rat taste buds contain higher percentages of PLCβ2-LIR cells than mouse taste buds (p < 0.001) (Fig. 2).

### Synaptobrevin-2

**Figure 6:**
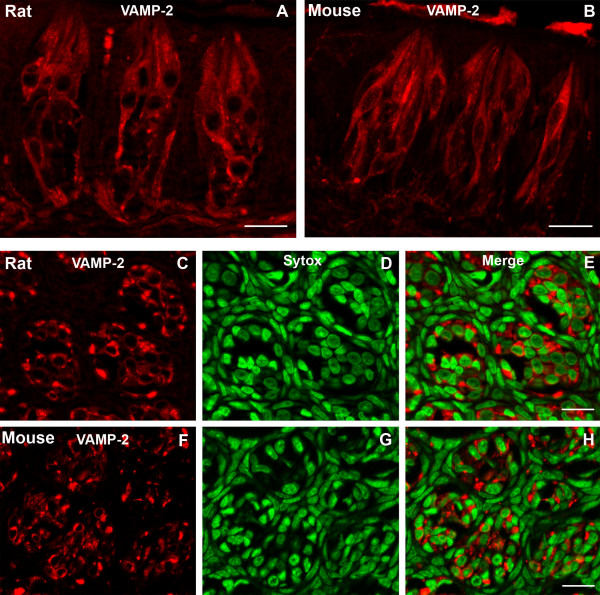
Confocal laser scanning microscopy (CLSM) images of synaptobrevin-2-LIR (VAMP-2) in taste buds of rat or mouse circumvallate papillae. Longitudinal sections show a large subset of taste cells and nerve processes displaying synaptobrevin-2-LIR in rat (A) and mouse taste buds (B). Transverse sections show synaptobrevin-2-LIR in rat (C-E) and mouse taste buds (F-H). The red are immunoreactive taste cells and nerve processes (C and F). Immunoreactivity is restricted to the cytoplasm (red) in both transverse and longitudinal sections. Sytox-stained nuclei (green) are shown in D and G and merges are shown in E and H. Scale bars = 20 μm.

Synaptobrevin-2 (VAMP-2) is a synaptic vesicle membrane protein that plays an important role in the exocytosis of neurotransmitter release at the synapse [38-40]. Previous studies have shown that synaptobrevin-2-LIR is present subsets of both type II and type III taste cells in rat taste buds [14]. Synaptobrevin-2 is present in a large subset of taste cells and nerve processes in both rat and mouse circumvallate taste buds (Fig. 6). Approximately 35% of the cells in taste buds from rat circumvallate papillae display synaptobrevin-2-LIR [14]. Most of the immunoreactive taste cells are spindle shaped with circular to ovoid nuclei. A smaller subset of synaptobrevin-2-LIR taste cells possessed cells that are slender in shape. We examined a total 152 taste buds from five rats and 241 taste buds from ten mice. We found 870 taste cells displaying synaptobrevin-2-LIR in rat circumvallate taste buds, and 1290 taste cells displaying synaptobrevin-2-LIR in mouse circumvallate taste buds (Table 1). There is a significantly higher percentage of taste cells displaying immunoreactivity to synaptobrevin-2 in rat circumvallate taste buds versus mouse taste buds (31.2% vs 26.3%) (Fig. 2).

### Numerical density of taste cells

Forty-one taste buds from 3 mice and 42 taste buds from 3 rats were analyzed (Table 2). Mouse taste buds contain an average of 85.8 taste cells (Mean ± SD: 85.8 ± 33.9) vs 68.4 taste cells (Mean ± SD: 68.4 ± 20.7) in rat taste buds. The numbers of cells per taste bud ranged from 32 to 152 in the mouse and 34 to 126 in the rat. Although the average mouse taste bud contains more taste cells than a rat taste bud, the average volume of a mouse taste bud (42,000 μm^3^) is smaller than a rat taste bud (64,200 μm^3^). The numerical density of taste cells in mouse circumvallate taste buds (2.1 cells/1000 μm^3^) is significantly higher than that in the rat (1.2 cells/1000 μm^3^)(Table 2).

**Table 2:** Numerical density of taste cells in rat and mouse circumvallate papillae taste buds

**Animals**	**No. of TB**	**Cell No./TB (Mean ± SD)**	**TB Volume (x1000 μm^3^) (Mean ± SD)**	**Density(N/1000 μm^3^) (Mean ± SD)**
Mouse 1^#^	13	69.2 ± 23.5	30.5 ± 10.5	2.28 ± 0.25
2^#^	15	105.3 ± 32.3	52.7 ± 18.5	2.03 ± 0.16
3^#^	13	79.8 ± 35.4	41.3 ± 19.8	1.98 ± 0.26
***Sum***	***41***	***85.8 ± 33.9***	***42.0 ± 18.9***	***2.10 ± 0.26***
Rat 1^#^	10	59.1 ± 16.5	34.9 ± 12.2	1.78 ± 0.38
2^#^	15	70.1 ± 16.9	66.5 ± 19.2	1.10 ± 0.26
3^#^	17	72.4 ± 24.8	79.5 ± 38.8	0.98 ± 0.26
***Sum***	***42***	68.4 ± 20.7*	64.2 ± 32.5*	1.20 ± 0.42*

*t-Test, p < 0.05, showing significant difference compared with the results of mice. TB = Taste Bud.

## Discussion

In the present study we have demonstrated that significant differences exist between rats and mice with regard to the presence of signaling molecules and taste bud cell markers. Using unbiased systematic sampling and immunocytochemistry we have quantified the presence of signaling molecules/taste cell markers including serotonin, PGP 9.5, α-gustducin, phospholipase C β2 (PLCβ2) and synaptobrevin-2. Our results indicate that there are significant differences (p < 0.05) between mouse and rat taste buds in the percentages of taste cells displaying immunoreactivity (IR) for all five markers. Higher percentages of rat taste bud cells exhibit immunoreactivity to α-gustducin, PLCβ2 and synaptobrevin-2 compared with the mouse. Mouse taste buds however, contain higher percentages of taste cells displaying serotonin- and PGP 9.5-LIR.

### Serotonin

Serotonin is a putative neurotransmitter or neuromodulator candidate in the taste bud [41,42]. Previous studies have suggested that serotonin is present in type III taste cells in rat, rabbit, and mouse taste buds [23,43,44]. Yee et al. [11] proposed that the type III cells in rat circumvallate taste buds are two of varieties: those immunoreactive for serotonin and those immunoreactive for PGP 9.5. Taste bud synapses in rat circumvallate taste buds are only associated with the type III cells [11,13,14]. Our quantitation results indicate there is a significant difference (p < 0.05) in the percentages of taste cells displaying serotonin-LIR between mouse and rat circumvallate taste buds: 15.9% of mouse taste cells contain serotonin compared with 13.7% of rat taste bud cells. Based on previous work from our laboratory, we believe that serotonin-LIR colocalizes with SNAP-25-LIR in taste cells of rat taste buds [45].

### PGP 9.5

PGP (protein gene product) 9.5 is a neuronal marker that has also been found in certain types of paraneurons [46,47]. PGP 9.5-LIR has been identified in taste buds of the rat [48,49]. Previously we found PGP 9.5-LIR in subsets of both type II and type III cells in circumvallate taste buds of the rat [11]. We also observed synapses onto nerve processes from PGP 9.5-LIR type III taste cells. Whereas one subset of type III cells in the rat accumulate serotonin but do not express PGP 9.5, the remainder of the type III cells express PGP 9.5 but do not accumulate serotonin. Similarly, two subsets of type II cells exist: those immunoreactive for PGP 9.5 and those immunoreactive for α-gustducin. Our results indicate that 14.3% of taste cells express PGP 9.5 in mouse, while 9.4% display PGP 9.5-LIR in rat. Thus, the PGP 9.5-LIR subsets of type II and type III cells may constitute small percentages of those cell types. It would be of benefit for future studies to elucidate the percentages of these subsets of type II and type III cells.

### α-gustducin and PLCβ2

α-gustducin and PLCβ2 are believed to participate in bitter, sweet and umami taste transduction [34-37]. α-gustducin knockout mice show markedly reduced behavioral and electrophysiological responses to both bitter and sweet compounds [31]. We have demonstrated that all α-gustducin immunoreactive cells and most PLCβ2-immunoreactive taste cells are type II taste cells. A small percentage (3.5%) of PLCβ2-immunoreactive taste cells appear to be type III cells [19,21]. Virtually all α-gustducin-LIR taste cells display PLCβ2-LIR, while only a subset of PLCβ2-immunoreactive taste cells display α-gustducin-LIR [20,22]. The percentages of α-gustducin- and PLCβ2-LIR taste cells in rat circumvallate taste buds (18% and 31.8% respectively) are higher than those in mouse (14.6% and 19.6%).

### Synaptobrevin-2

Synaptobrevin-2 is a vesicle-associated membrane protein. Previous results from our laboratory indicate that synaptobrevin-2 is present in a subset of type II and type III cells. Our data suggest that taste cells with synapses express synaptobrevin-2 [14]. In rat circumvallate taste buds, a large subset of synaptobrevin-2-LIR cells (73%) also express IP_3_R3 [14]. Most all IP_3_R3 immunoreactive cells have been shown to be type II cells [21]. In the present study we have found that a greater percentage of rat taste cells display immunoreactivity for synaptobrevin-2 versus the mouse (31.2% vs 26.3%). Likewise, rats have a larger percentage of taste cells expressing α-gustducin and PLCβ2. These findings suggest that proportionally there are more type II cells in rat circumvallate papillae taste buds when compared with mouse. Although type II taste cells lack classical synapses, we do find that the type II taste cells contain some vesicles in the cytoplasm. The function of synaptobrevin-2 in type II taste cells is unclear, however, it suggests that synaptobrevin-2 may play a role in vesicle protein transportation, perhaps in the Golgi apparatus.

Several investigators have used different immunohistochemical methods to quantify taste cells displaying α-gustducin or PGP 9.5 in rodent animals. Ueda et al. [25] used the avidin-biotin-horseradish peroxidase (ABC) method and concluded that approximately 24.2% of rat circumvallate papillae taste bud cells display α-gustducin-LIR and 14.6% display PGP 9.5-LIR. The results in that study were based on 320 taste cells in 20 taste buds. This contrasts with our results from the rat (α-gustducin, 18%; PGP 9.5, 9.4%). This disparity may be due to: 1) The number of taste buds we sampled (α-gustducin: 197 taste buds; PGP 9.5: 144 taste buds in the present study versus approximately 20 taste buds by Ueda et al. [25]); 2) Our use of unbiased sampling; 3) Specimen preparation techniques, e.g., the use of different fixatives; 4) Immunocytochemical imaging methods e.g., ABC method vs immunofluorescence. Smith et al. [50] reported that rat circumvallate taste buds have a mean of 8.37 α-gustducin-LIR cells per taste bud. Takeda et al. [26] found α-gustducin-LIR in 33% and PGP 9.5-LIR in 23% of mouse circumvallate taste bud cells. We account for the difference in our results for the following reasons: 1) We used unbiased systematic sampling in our study; 2) We analyzed over 140 taste buds for each antibody; 3) In our study, taste cells were counted as immunoreactive only when a nuclear profile was present; 4) We counted immunoreactive taste cells using transverse sections versus longitudinal sections. In the transverse sections, there is no overlapping in taste cells, the immunoreactive taste cell profiles are obvious, and nuclei are easier to count. Takeda et al. [26] used polyclonal PGP 9.5 antibody in their study while we used a monoclonal PGP 9.5 antibody. However, our experience with polyclonal PGP 9.5 (Code No. 7863-0507, Biogenesis) is that it completely colocalizes in taste cells and nerve processes with monoclonal PGP 9.5 antibody (Code No. 7863-1004, Biogenesis). Finally, we conclude that a higher percentage of rat taste cells express α-gustducin (18%) than in the mouse (14.6%); while a smaller percentage of rat taste cells express PGP 9.5 (9.4%) versus the mouse (14.3%).

### Numerical density and size of taste buds

It is generally accepted that a rodent taste bud contains 50 – 150 taste cells. We were curious to determine if there are differences in the numbers of cells in circumvallate taste buds between the rat and mouse. Our results clearly demonstrate that mouse taste buds are smaller in volume, but contain a larger number of smaller taste cells when compared with rat.

## Conclusion

We have provided evidence that the rat and mouse differ in the percentages of taste cells expressing each of five taste signaling molecules: serotonin, PGP 9.5, α-gustducin, PLCβ2 and synaptobrevin-2. These results, taken together with the differences taste cell size and numbers, suggest that rats and mice may possess different sensitivities to gustatory stimuli.

## Methods

Adult Sprague-Dawley male rats (250–350 g, 45 days) and CF-1 male mice (25–30 g, 49 days) purchased from Charles River were used for these studies. Animals were cared for and housed in facilities approved by the Institutional Animal Care and Use Committee of the University of Denver. For studies involving serotonin, animals were injected with 5-hydroxytryptophan (5-HTP, 80 mg/kg, i.p.) one hour before sacrifice. All animals were anesthetized with ketamine HCI about 270 mg/kg body weight for rats and 370 mg/kg body weight (i.p.) for mice. Animals were perfused for 10 seconds through the left ventricle with 0.1% sodium nitrite, 0.9% sodium chloride and 100 units sodium heparin in 100 ml 0.1 M phosphate buffer (pH 7.3). This was followed by perfusion with 4% paraformaldehyde in 0.1 M phosphate buffer for 10 minutes [51]. All perfusates were warmed to 42°C before use. After perfusion the excised circumvallate papillae were fixed in fresh fixative for 3 hours at 4°C. The tissues were cryoprotected with 30% sucrose in 0.1 M phosphate buffer overnight at 4°C.

### Unbiased systematic sampling method

Five adult Sprague-Dawley male rats and ten CF-1 male mice were perfused as for immunohistochemistry. Serial transverse sections (20 μm thickness) were cut from the tissues containing circumvallate taste buds using a cryostat (HM 505E, MICRON, Laborgeräte GmbH, Germany). In order to obtain a systematic sample without bias throughout the papilla, each papilla was exhaustively sectioned. The serial sections were placed sequentially into individual wells in a 36-well culture dish. Every fifth section was saved starting with section 1, 2, 3, 4, or 5. The beginning section number was determined using a new random number for each rat (e.g., sections 3, 8, 13, 18, and 23). Assuming that a taste bud is 80–100 μm in length, sampling every fifth section will assure that no two sections will be from the same taste bud. Each group of sections contains 25–30 sections from five rat circumvallate papillae. For the sections from the mouse circumvallate papilla, every third section was saved using the sampling method described above.

### Immunofluorescence and nuclear staining

Cryostat sections were blocked in 5% normal goat serum and 0.3% Triton X-100 in 0.1 M phosphate buffered saline (PBS) (pH 7.3) for one hour at room temperature, followed by incubation in a primary antibody (Table 3) in 0.1 M PBS (pH 7.3) overnight at 4°C. After washing, the sections were exposed to affinity-purified secondary antibody Cy5 conjugated to goat anti-rabbit IgG (diluted to 1:200, cat no. 111-175-144, Jackson Lab) in 0.1 M PBS (pH 7.3) for one hour at room temperature. In order to image the nuclei the sections were stained using Sytox green nucleic acid stain (S-7020, Molecular Probes, Eugene, OR)

**Table 3:** Primary antibodies

**Antibodies**	**Species**	**Dilution**	**Source**	**Cat No.**
Serotonin	Rabbit	1:100	ImmunoStar	20080
PGP 9.5	Rabbit	1:200	Biogenesis	7863-0507
α-gustducin	Rabbit	1:200	Santa Cruz	sc-395
PLCβ2,	Rabbit	1:200	Santa Cruz	sc-206
Synaptobrevin-2	Rabbit	1:100	Wako	018-15791

#### Controls

Primary antibodies were excluded from the processing to check for cross-reactivity. No immunoreactivity was observed under these conditions.

### Quantification of immunoreactive taste cells

Confocal images were collected using a Zeiss Axioplan II with an Apotome attachment (Carl Zeiss Advanced Imaging Microscopy, Germany). Approximately 140–200 rat taste buds and 150–240 mouse taste buds per group were analyzed. Cells were scored as immunoreactive only if a nuclear profile was present in the cell. The total number of cells in the slice was determined by counting the number of Sytox stained nuclei for each taste bud. Finally, the percentage of immunoreactive taste cells was calculated by dividing the number of immunoreactive taste cells by the total number of the taste cells in each taste bud.

### Determination of numerical density of taste cells in rat and mouse taste buds

After perfusion, the excised circumvallate papillae were fixed with fresh fixative for 3 hours at 4°C. The tissues were then postfixed and stained for two hours in 1% osmium tetroxide (OsO_4_) in 0.1 M PO_4 _buffer followed by a rinse in 0.05 M sodium maleate buffer (pH 5.2). The blocks were then stained en bloc in 1% uranyl acetate in 0.025 M sodium maleate buffer (pH 6.0) overnight at 4°C, followed by dehydration and embedding in Eponate 12. The blocks were the re-embedded using the technique of Crowley and Kinnamon [52].

Serial thin sections (1 μm) were cut with a Diatome Histo-Jumbo Knife using a Leica Ultracut UCT Ultramicrotome. Typically a ribbon of about 20 sections was collected onto a glass slide. After drying on a hot plate the sections were stained with toluidine blue for 5 minutes. Images of taste buds were recorded using a Zeiss Axioplan II with an Apotome attachment. The images of taste buds were collected from every other section. Using Adobe Photoshop we compared every two adjacent images and identified the number of newly occurring taste cell nuclei. The number of taste cells in a taste bud was the sum of newly occurring taste cell nuclei that appeared in every other image in the series.

The volume of a taste bud was calculated according to following formula: Volume (μm^3^) = Σ_1-n _(37.2 × 2 × C_n_) (C: number of crosses on taste bud image; n: image number; 2: the thickness is 2 μm between two adjacent images). We superimposed an image of grids (20 × 20 grids, 1 cm/grid) over the image of a taste bud profile and counted the number of crosses within a taste bud profile. Each cross represents an area of 37.2 μm^2^. Every taste bud area was multiplied by the thickness between two adjacent sections and summed to determine the volume of the section. The volumes of all of the sections were summed to obtain the volume of the taste bud.

Numerical density of taste cells in a taste bud was calculated by dividing the number of taste cells by the volume of the taste bud.

### Statistical analysis

Statistical analysis for the percentages of immunoreactive taste cells in Figure 2 and the numerical density of taste cells in Table 2 were performed using the Student *t*-test.

## Authors' contributions

HM, RY, and JCK participated in the study design. HM carried out the animal experiments. The quantitation data were collected and analyzed by HM, RY, and ST. HM drafted the manuscript. The study was conceived and funded by JCK. All authors participated in the writing of and approved the final manuscript.
